# Advancing Non-Invasive Prenatal Screening: A Targeted 1069-Gene Panel for Comprehensive Detection of Monogenic Disorders and Copy Number Variations

**DOI:** 10.3390/genes16040427

**Published:** 2025-04-02

**Authors:** Roberto Sirica, Alessandro Ottaiano, Luigi D’Amore, Monica Ianniello, Nadia Petrillo, Raffaella Ruggiero, Rosa Castiello, Alessio Mori, Eloisa Evangelista, Luigia De Falco, Mariachiara Santorsola, Michele Misasi, Giovanni Savarese, Antonio Fico

**Affiliations:** 1 Centro AMES, 80013 Casalnuovo di Napoli, Italy; ced@centroames.it (L.D.); monica.ianniello@centroames.it (M.I.); nadia.petrillo@centroames.it (N.P.); raffaella.ruggiero@centroames.it (R.R.); rosa.castiello@centroames.it (R.C.); giovanni.savarese@centroames.it (G.S.); centroames@libero.it (A.F.); 2 Istituto Nazionale Tumori di Napoli, IRCCS Fondazione Pascale, Via M. Semmola, 80131 Naples, Italy; a.ottaiano@istitutotumori.na.it (A.O.); mariachiara.santorsola@istitutotumori.na.it (M.S.); 3 Department of Gynecology and Obstetrics, Universiteti Katolik Zoja e Këshillit të Mirë, Rr. Dritan Hoxha, 1057 Tirane, Albania

**Keywords:** non-invasive prenatal screening, circulating-free fetal DNA, monogenic disorders, copy number variations, next-generation sequencing

## Abstract

We introduce an innovative, non-invasive prenatal screening approach for detecting fetal monogenic alterations and copy number variations (CNVs) from maternal blood. Method: Circulating free DNA (cfDNA) was extracted from maternal peripheral blood and processed using the VeriSeq NIPT Solution (Illumina, San Diego, CA, USA), with shallow whole-genome sequencing (sWGS) performed on a NextSeq550Dx (Illumina). A customized gene panel and bioinformatics tool, named the “VERA Revolution”, were developed to detect variants and CNVs in cfDNA samples. Results were compared with genomic DNA (gDNA) extracted from fetal samples, including amniotic fluid and chorionic villus sampling and buccal swabs. Results: The study included pregnant women with gestational ages from 10 + 3 to 15 + 2 weeks (mean: 12.1 weeks). The fetal fraction (FF), a crucial measure of cfDNA test reliability, ranged from 5% to 20%, ensuring adequate DNA amount for analysis. Among 36 families tested, 14 showed a wild-type genotype. Identified variants included two deletions (22q11.2, and 4p16.3), two duplications (16p13 and 5p15), and eighteen single-nucleotide variants (one in *CFTR*, three in *GJB2*, three in *PAH,* one in *RIT1*, one in *DHCR7*, one in *TCOF1*, one in *ABCA4*, one in *MYBPC3*, one in *MCCC2*, two in *GBA1* and three in *PTPN11*). Significant concordance was found between our panel results and prenatal/postnatal genetic profiles. Conclusions: The “VERA Revolution” test highlights advancements in prenatal genomic screening, offering potential improvements in prenatal care.

## 1. Introduction

The advent of prenatal genetic diagnosis marks a significant milestone in modern obstetrics, revolutionizing the early detection and management of genetic and genomic disorders. The last decade has seen a dramatic development of sequencing techniques widening the spectrum of possible diagnosis with this technology. In light of these technical advancements, monogenic disorders, resulting from alterations in a single gene, often manifest as rare yet medically significant challenges, have been thoroughly investigated. Despite their low prevalence, typically affecting fewer than 5 in 10,000 individuals, the implications of monogenic diseases on affected families and their progeny are profound, often necessitating comprehensive clinical interventions [1]. Traditional prenatal diagnostic techniques, such as amniocentesis and chorionic villus sampling (CVS), have long been recognized as reliable methods for detecting chromosomal and genetic abnormalities. However, as invasive procedures, they carry inherent risks, including miscarriage and infection [2]. In contrast, non-invasive approaches, while significantly reducing procedural risks, remain screening tools rather than definitive diagnostic tests, necessitating further confirmatory testing when a high-risk result is identified. Lo et al. [3] first reported the presence of fetal DNA in maternal plasma, laying the groundwork for prenatal cell free DNA screening. The detection of cell-free fetal DNA (cfDNA) circulating in maternal blood has catalyzed a paradigm shift towards non-invasive prenatal screening (NIPS). The implementation of NIPS leverages state-of-the-art advancements in genomic technologies, notably next-generation sequencing (NGS) and digital polymerase chain reaction (dPCR) [4]. These methodologies facilitate the enumeration and analysis of cfDNA fragments, enabling the detection of genetic abnormalities from early stages of gestation. Consequently, NIPS offers prospective parents’ critical insights into the genetic health of their offspring, empowering timely clinical decision-making and intervention strategies. Notwithstanding the promise of NIPS, the accurate diagnosis of monogenic disorders through this procedure poses substantial challenges. For common autosomal dominant disorders, a cfDNA noninvasive pre-natal diagnostic approach has also been developed through sequencing of a panel of causative genes [5]. A predominant obstacle mainly in recessive condition is the differentiation of the circulating fetal DNA from the abundant maternal circulating DNA that requires more sensitive methods to accurately detect small imbalances between the amount of the wild type and pathogenic allele in maternal plasma, given that the fetal genotype is present with a high background of maternal cfDNA. However, recent strides in molecular diagnostics and bioinformatics have significantly improved the diagnostic capabilities of NIPS. Through the refinement of sequencing technologies and the development of sophisticated algorithms for data analysis, the NIPS has been given the possibility to go further the genomic level of analysis and to give essential and specific insights in the fetal genome of the fetus [6,7,8]. Currently, cfDNA noninvasive prenatal diagnosis for monogenic disorders is technically possible and available in a number of countries, but its use is largely limited to pregnancies at increased risk [9]. The use of the cfDNA to screen for common monogenetic conditions in low-risk pregnancies in clinical practice is still under debate [10]. To date, there are few studies with complete follow-up of pregnancies screened for CNVs, and sensitivity and specificity data are limited [11]. In fact, the current ACMG position statement did not recommend NIPS for genome-wide CNV screening, primarily because of the limited clinical utility and uncertainties regarding PPV and NPV and the lack of clinical validation of routine use [12]. In this study, we aim to validate the detection accuracy, efficiency, and clinical utility of an innovative approach to NIPS through a genetic test based on a combination of an expanded gene panel and new bioinformatic analysis, capable of screening for conditions associated with both monogenic alterations and copy number variations (CNVs). Our test applies the most comprehensive spectrum of single nucleotide polymorphisms (SNPs) and CNVs analysis currently available for NIPS. The objective was to determine if deep sequencing of cfDNA could detect pathogenic variants and identify paternal and de novo variants in the fetus by comparing them to those identified in the amniotic fluid, CVS, or newborn samples.

## 2. Materials and Methods

### 2.1. Patient Recruitment and Sample Processing

A total of 36 singleton pregnancies were enrolled in this study at Centro Ames (Naples, Italy) following fully informed consent from the participants. We analyzed 144 DNA specimens, including 36 cell-free fetal DNA samples, 72 genomic DNA samples from parents and 36 genomic DNA from amniotic fluid (AF), chorionic villus sample (CVS) or buccal swabs. The families included in this study were recruited over a one-year period, from May 2023 to May 2024, and were referred for genetic counseling and assessment either due to being at risk (based on suspected ultrasound findings or positive family history) or by personal request for NIPS. The study was conducted according to the guidelines of the Declaration of Helsinki and a strong emphasis on informed consent. Prior to sample collection, all participants received detailed information about the study’s objectives, the nature and procedures of the genetic testing, potential risks and benefits, and the confidentiality of their genetic data. Sample collection was then performed according to the established protocols.

### 2.2. DNA Isolation and NIPS Analysis

Genomic DNA (gDNA) was extracted and isolated from peripheral blood in EDTA, buccal swabs, andCVS using the MagCore Nucleic Acid Extraction Kit (Diatech Pharmacogenetics, Jesi, Italy), while gDNA from AF was extracted and isolated using the QIAamp^®^ DNA Blood Mini Kit (Qiagen, Hilden, Germany), and according to the manufacturer’s guidelines and instructions. For circulating-free DNA (cfDNA) extraction and isolation, a total of 10 mL of peripheral blood was collected from pregnant women using Streck blood collection tubes. The blood samples were centrifuged at 1600× *g* for 10 min at 4 °C to separate plasma from peripheral blood cells. CfDNA was then extracted from 900 µL of maternal plasma using the QIAamp^®^ DNA Blood Mini Kit (Qiagen, Hilden, Germany) through the QIAvac Plus Vacuum Manifold system, following the manufacturer’s protocol. CfDNA was first used for NIPS analysis, performed with a pipeline involving automated plasma separation, cfDNA extraction and isolation, library preparation using the VeriSeq NIPT Solution v2 (Microlab STAR, Illumina, San Diego, CA, USA) and shallow whole-genome sequencing (sWGS) on a NextSeq550Dx (Illumina), according the VeriSeq NIPT Solution v2 package insert (available online at https://support.illumina.com/downloads/veriseq-nipt-solution-v2-package-insert-1000000078751.html, last accessed on 1 July 2024). The concentration and purity of the isolated DNA were assessed using spectrophotometry and fluorometry, which provided accurate measurements of cfDNA concentration.

### 2.3. VERA Revolution Gene Panel: Design and Selection Criteria

The custom gene panel used, named “VERA Revolution”, consists of 1069 genes providing comprehensive coverage for a wide range of monogenic disorders showed in the Supplementary Table S1. Genes were selected based on several criteria to encompass a broad spectrum of genetic disorders with significant clinical relevance. The conditions included Noonan spectrum disorders, skeletal disorders, craniosynostosis syndromes, Cornelia de Lange syndrome, Alagille syndrome, tuberous sclerosis syndrome, epileptic encephalopathy, SYNGAP1-related intellectual disability, CHARGE syndrome, Sotos syndrome, Rett syndrome and cardiomyopathies. The panel addresses conditions with varying incidences, from common disorders affecting approximately 1 in 2000 individuals to rare diseases with incidences as low as 1 in 100,000 or less (Table S1)

In the setup procedure for the choice of genes and diseases, priority was given to genes with well-documented associations with diseases that have substantial health impacts. The selection process involved a thorough review of current literature and clinical genetic databases such as ClinVar and OMIM to ensure the inclusion of clinically significant genes. 

The panel encompasses genes associated with various inheritance patterns, including autosomal dominant, autosomal recessive, X-linked disorders. The diversity allows the panel to be applicable in a wide range of genetic testing scenarios.

The protein function and molecular mechanism of each gene also play a crucial role in its inclusion. The panel incorporates genes involved in metabolic pathways, ion transport, DNA repair, structural integrity, transcription regulation, and mitochondrial function, reflecting the diversity of genetic mechanisms underlying human disorders. This selection ensures that the panel can effectively detect conditions caused by enzyme deficiencies, protein misfolding, or impaired cellular signaling.

In addition, genes were chosen for their potential to enhance diagnostic yield during prenatal screening. We included genes where mutations are known to cause highly penetrant disorders, making early detection critical for effective patient management and improved outcomes. Some genes were included based on the capabilities of Next Generation Sequencing (NGS) to detect complex mutations, such as large deletions, duplications, or rearrangements, which may not be identified by other technologies.

### 2.4. VERA Revolution Library Preparation

Genomic DNA from peripheral blood collected in EDTA, buccal swabs, chorionic villus sampling (CVS) and amniotic fluid, and cfDNA from maternal plasma were extracted as aforementioned. According to KAPA HyperPlus kits (Kapa Biosystems, Roche Diagnostics, Wilmington, MA, USA) manifacturer’s instructions, first of all, gDNA was fragmented to achieve the size required by the applied Illumina short-reads sequencing technology. According to KAPA HyperPrep kits (Kapa Biosystems, Roche Diagnostics, Wilmington, MA, USA) and given the fragmentation of cfDNA, no additional fragmentation steps were required for the sample extracted from maternal plasma. Fragmented gDNA and cfDNA underwent to simultaneous library preparation steps of end-repair and A-tailing, followed by a 15 min ligation process with Unique Dual-Indexed (UDI) and an 18 h ligation process with Unique Molecular Identifiers (UMI) adapters, respectively. UDI adapters are required for fragments interaction with flow-cell surface during the sequencing process while UMIs use aids in accurate molecule tracking and error correction. After UDI and UMIs ligation, the first purification phase removed excess unbound reagents to reduce background noise. The protocol continued with the annealing of UDI Primer Mixes, essential for effective multiplexing during high-throughput sequencing. This step ensures selective amplification of target sequences and minimizes index hopping. Following UDI primer attachment, a second purification for gDNA and double size selection for cfDNA were made to isolate DNA fragments within the desired size range, optimizing library quality and sequencing consistency. Probes manufactured by Roche (Kapa Biosystems, Roche Diagnostics, Wilmington, MA, USA) were used for capturing the genomic regions specified in the custom gene panel. Hybridization using custom-designed oligonucleotide probes was performed for up to 20 h to optimize both the capture efficiency and specificity of target sequences. Following hybridization, a purification step was implemented to enrich for the desired DNA fragments. Subsequently, the enriched library was amplified using random primers to increase the total quantity of DNA while preserving sequence diversity. Finally, the amplified products were pooled and prepared for next-generation sequencing, targeting specific genomic regions. This multi-step approach aims to achieve high-fidelity sequencing data for both genomic DNA (gDNA) and cell-free DNA (cfDNA), thereby supporting reliable diagnostic applications and genomic research. Data analysis for SNV involved alignment using Isaac Genome Alignment Software version 4 and variant calling with GATK v.4.2.0.0, ISAAC v.1.0.7, and LoFreq v.2.1.2 to cover the full range of identified variant frequencies. As illustrated in the manuscript, variant comparison was conducted between those identified with GATK and ISAAC in the born child versus those detected by LoFreq in the cfDNA. Variants in the born child were pre-filtered to exclude artifacts. The primary filter excluded variants with allele frequencies at or below 20% or above 80%, as these might be enriched with artifacts. The second filter excluded regions of low coverage mapability and repeats, as listed in the ENCODE database [13]. Variants with a read depth less than 100× were removed via a third filtering step. Following artifact filtration, a trio-based analysis, utilizing the father, mother, and proband, was conducted to discern paternally inherited variants and de novo mutations by examining alleles in the proband that were not present in the maternal genome and were absent in both parental genomes, respectively.

### 2.5. CNV Calling

We developed a combined approach utilizing two robust methodologies to detect variants and copy number variations (CNVs) in cfDNA samples. The rationale behind this combined approach was to improve the performance of prenatal genetic screening by integrating the advantages of each methodology, thereby promoting more accurate and reliable results. The first methodology employed is based on a well-established approach for detecting single nucleotide variants (SNVs) in mixed DNA samples, such as cell-free DNA (cfDNA), where fetal DNA is present within a background of a higher proportion of maternal DNA. This method utilizes an allele frequency-based model to predict the presence of variants, particularly in low-frequency settings [14]. The model assumes a binomial distribution of allele counts, where observed counts at a given SNP site are used to estimate the probability of different genotypes (homozygous reference, heterozygous, or homozygous variant). It is particularly robust in scenarios with low fetal DNA fractions, enabling the identification of variants that might otherwise be obscured by the predominance of maternal DNA. We implemented this method as the initial step in our analytical pipeline, not as an end in itself, but rather to specifically identify fragments likely originating from the fetal genome. This allows for the selective focusing on these fragments for subsequent CNV analysis. For CNV detection, we adopted a second methodology that builds upon the data obtained from the initial SNV analysis, focusing on the analysis of DNA fragment sizes. This method estimates CNVs by analyzing the distribution of fragment sizes in the cfDNA [15]. We implemented it following the initial SNV analysis. By basing our CNV estimation specifically on these identified fetal-derived fragments, we were able to bypass the direct analysis of all cfDNA fragments. This strategy enabled efficient detection of deletions or duplications in the fetal genome. The method starts by modeling the pure distribution of fetal fragment sizes using the SNV data to estimate the size distribution of fragments mapped to targeted regions and compare them to reference control bins. Maximum likelihood estimation is then applied to predict CNVs within the targeted genomic regions, based on deviations from the expected normal distribution. More details on the analysis method will be provided upon request to the corresponding author (Table S2).

### 2.6. Follow-Up Diagnostic Testing

Follow-up has been performed for all investigated cases both for positive and negative ones, tests were carried out in the AMES laboratories at no additional cost. Follow-up data were entered into a database. Clinical truth was determined via invasive testing (chorionic villus sampling or amniocentesis), or the use of buccal swab at birth. Amniotic fluid was drawn, and genomic DNA was extracted from the amniocyte using the QIAamp DNA Blood Mini Kit (Qiagen). Genomic DNA was extracted using peripheral blood in EDTA, buccal swabsand CVS according to the manufacturer’s instructions (MagCore Nucleic Acid Extraction Kit Diatech Pharmacogenetics, Jesi, Italy). For karyotype analysis, GTG-banding analysis was performed in established cell culture following standard laboratory protocols. Metaphases were analyzed with the CytoVision software version 7.6 (CytoVision, AB Imaging, Kuala Lumpur, Malaysia). SNP array analysis was performed using a Infinium™ Global Screening Array-24 v3.0 BeadChip kit, which also allowed testing for the presence of a uniparental disomy, following the manufacturer’s protocol (https://www.illumina.com/products/by-type/microarray-kits/infinium-global-screening.html accessed on 1 February 2023). Stained Bead-Chips were scanned with a HiScan™SQ System (Illumina, Inc., San Diego, CA, USA). Data were generated with GenomeStudio software (Illumina, Inc.) and analyzed with the NxClinical (Bionano, San Diego, CA, USA). All CNVs > 100 Kb were interrogated. All results were reported according to the GRCh37 (hg19) assembly [16].

## 3. Results

### 3.1. VERA Revolution Gene Panel and Depth of Analysis

**Figure 1:**
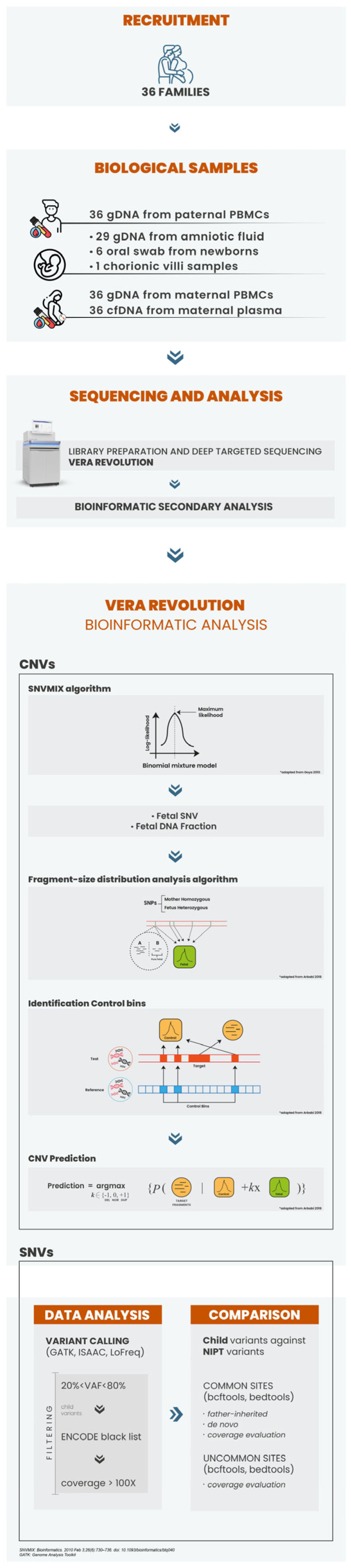
Workflow for establishing the VERA Revolution procedure [14].

The workflow for developing the gene panel analysis is depicted in Figure 1. The final version of our genetic panel includes a total extent of 3,490,181 bp (base pairs) and 18,583 capture targets for SNPs, which strikes a balance between its size and its feasibility in both technical and economic terms. To further enhance the utility of the panel, it is enriched with probes specifically designed to cover over 100 microdeletion syndromes and, where applicable, duplications events. Each targeted region is selected based on its clinical relevance, as evidenced by its inclusion in databases such as OMIM (Online Mendelian Inheritance in Man) and DECIPHER (Database of Chromosomal Imbalance and Phenotype in Humans using Ensembl Resources). The custom microdeletion panel covers a total genomic extent of approximately 1,523,296,268 bp across all the loci analyzed. On average, each region within the panel spans approximately 16,205,279 bp. The analyzed cases revealed regions of both duplications and deletions across multiple chromosomes, with sequencing depths ranging from approximately 53,000 to 238,000 reads on average covering the target regions. This broad approach ensures robust genetic analysis, enabling detailed genetic screening in a clinical setting.

**Figure 2:**
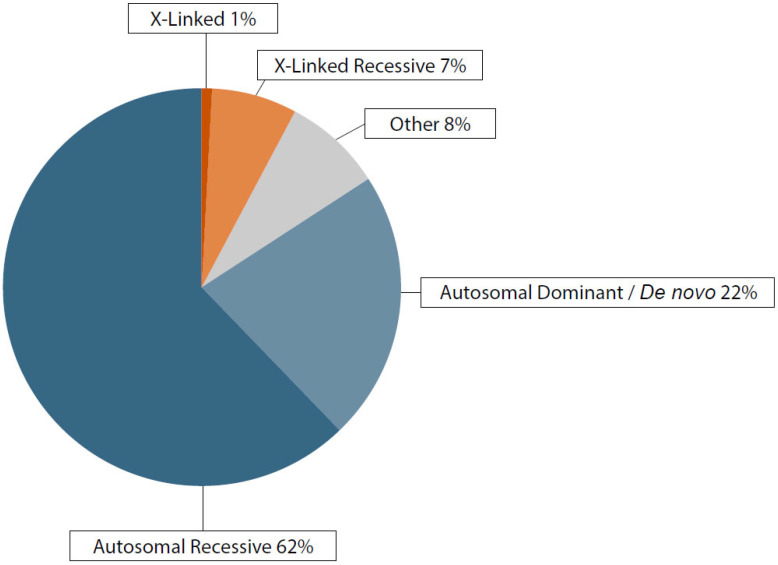
Frequencies of genetic inheritance patterns across the VERA Revolution panel for studying monogenic disorders.

The disorders covered by our custom panel, range in prevalence from 1 in 2000 to 1 in 100,000, encompassing conditions that can manifest at any stage of prenatal or postnatal development. Figure 2 displays a pie chart detailing the distribution of genetic inheritance patterns within the “VERA Revolution” panel for investigating monogenic disorders.

The upper panel illustrates the collection and analysis of biological samples from 36 families for prenatal genetic assessment. Samples were obtained from amniotic fluid, chorionic villi, newborn oral swabs, circulating free DNA (cfDNA) from maternal plasma, and genomic DNA (gDNA) from maternal and paternal peripheral blood mononuclear cells (PBMCs). CfDNA from maternal plasma was sequenced using the deep targeted sequencing technology (central panel). The bioinformatic analysis concentrated on identifying copy number variations (CNVs) (lower left panel) and single nucleotide variants (SNVs) (lower right panel), using custom algorithms to evaluate cfDNA fractions in the samples. Predictive modeling based on fragment size distribution and binomial mixture models was applied to assess genetic variations (lower left panel). Additionally, several variant calling techniques were employed to detect significant single nucleotide variants, which were compared to non-invasive prenatal screening (NIPS) results, with further evaluation of paternal inheritance and de novo variants (lower right panel).

The largest segment, comprising 62% of the chart and colored in blue, represents autosomal recessive (AR) disorders, underscoring the panel focus on conditions requiring two copies of the mutated allele for disease manifestation. This emphasis is crucial due to the complex nature of recessive conditions, where both parents must carry the allele for offspring to be affected. A notable 22% of the chart, shown in light blue, is allocated to autosomal dominant (AD) disorders, reflecting the significance of identifying conditions where a single defective gene copy from one parent leads to disease. This highlights the importance of dominant disorders in prenatal diagnostics. Smaller segments include a 7% portion in orange for X-linked recessive (XLR) disorders, which are important for understanding sex-linked conditions due to their X chromosome location. An additional 1% in red covers X-linked (XL) inheritance, emphasizing the complexity of these disorders. Finally, an 8% grey segment labeled ‘Other’ encompasses genetic conditions that do not fit neatly into the major categories but are critical for comprehensive genetic analysis, including conditions with unknown or poorly understood inheritance patterns.

### 3.2. Genetic Findings

Of the 300 families who visited the AMES center between January 2023 and January 2024, either due to a referral from their gynecologist for being at risk (advanced maternal age, family history of genetic disorders, or abnormal ultrasound findings) or by personal request for non-invasive prenatal screening (NIPS), 36 consented to undergo the “VERA Revolution” test. Table 1 presents the results of analyzing these 36 samples using the aforementioned test, compared to other diagnostic tests, including NIPS, NGS, and SNP array. The cohort comprised pregnant women at various gestational ages, ranging from 10 + 3 to 15 + 2 weeks with a mean of 12.1 weeks. The fetal fraction (FF), a key metric in the reliability of cfDNA tests, varied between 5% and 20% across the dataset, indicating sufficient DNA quantities for reliable genetic analysis in all cases. Out of the 36 families tested using our custom panel, 14 sample were negative both for SNV and CNV, these results were confirmed by orthogonal tests such as karyotype and SNP-array. Among positive samples we identified eighteen samples with SNV classified as pathogenic, two samples with deletions and two samples with duplications.

Samples 1, 11, 22 and 28 presented variants of the *GJB2* gene classified as pathogenic (Table 1). Three samples (ID 1, 11 and 28) showed the deletion of a single nucleotide, guanine (G), at position 35 (c.35delG; p.Gly12ValfsTer; NM_004004.6). Sample 1 has been found homozygous through NGS analysis with both parents being heterozygous for the mutation. Sample 11 has been found heterozygous for the paternal mutation p.Gly12ValfsTer through NGS analysis. The sample 22 carried two mutations, a missense mutation where valine at position 37 is replaced by isoleucine (c.109G>A; p.Val37Ile, NM_004004.6, rs72474224), and a second missense mutation where methionine at position 195 is substituted with valine (c.583A>G; p.Met195Val). The mutations were confirmed through NGS analysis where the p.Val37Ile has been found heterozygous in mother and p.Met195Val being heterozygous in father.

Sample 5 presented a homozygous pathogenic variant in *CFTR*. The c.1521_1523del, p.Phe508DelNM_000492.3 mutation in the *CFTR* gene involves the deletion of three nucleotides, resulting in the loss of phenylalanine (Phe) at position 508. This deletion leads to the misfolding of the CFTR protein, impairing its function.

Sample 12 presented a pathogenic variant in *RIT1*, the c.229G>T (p.Ala77Ser), NM_006912.6 mutation which results in a substitution of alanine (Ala) with serine (Ser) at position 77. This amino acid change is located within the GTP-binding domain of the RIT1 protein, which plays a crucial role in signal transduction. The Ala77Ser mutation has been associated with aberrant activation of downstream signaling pathways, leading to dysregulated cell proliferation and differentiation, which are implicated in Noonan syndrome and other developmental disorders.

Sample 25 presented a pathogenic variant in *TCOF1*. The c.1999dup (p.Arg667fs), NM_001371623.1 mutation involves the duplication of a nucleotide at position 1999, leading to a frameshift starting at arginine (Arg) 667. This frameshift mutation results in a premature termination codon, likely leading to nonsense-mediated mRNA decay or the production of a truncated TCOF1 protein. Given the role of TCOF1 in ribosome biogenesis and craniofacial development, this mutation is associated with Treacher Collins syndrome, a disorder characterized by craniofacial abnormalities due to defects in neural crest cell function.

Sample 26 presented a de novo pathogenic variant in *PTPN11*. The c.1471C>T (p.Pro491Ser), NM_002834.5 mutation results in a substitution of proline (Pro) with serine (Ser) at position 491. This amino acid change occurs within the SH2 domain of the PTPN11 protein, which is crucial for protein-protein interactions and signal transduction in the Ras/MAPK pathway. The Pro491Ser mutation has been associated with Noonan syndrome, a RASopathy characterized by a wide range of developmental abnormalities.

Sample 27 presented a pathogenic variant in *MCCC2*. The c.1015G>A (p.Val339Met), NM_022132.5 mutation leads to a substitution of valine (Val) with methionine (Met) at position 339. MCCC2 is a subunit of the 3-methylcrotonyl-CoA carboxylase enzyme, involved in the leucine degradation pathway. This mutation is associated with 3-Methylcrotonyl-CoA carboxylase 2 deficiency, a metabolic disorder that can present with various symptoms including hypotonia, developmental delay, and metabolic crises. NGS analysis confirmed the variant in the proband, and further analysis indicated that both parents were heterozygous for this mutation.

Sample 29 presented a pathogenic variant in *GBA1*. The c.1226A>G (p.Asn409Ser), NM_001005741.3 mutation results in a substitution of asparagine (Asn) with serine (Ser) at position 409 in the glucocerebrosidase enzyme. This enzyme is responsible for the breakdown of glucocerebroside. This mutation is a common cause of Gaucher disease type 1, a lysosomal storage disorder characterized by the accumulation of glucocerebroside in various organs. NGS analysis confirmed the heterozygous presence of this mutation in the proband, with the mother also carrying the same heterozygous mutation.

Sample 30 presented a de novo pathogenic variant in *PTPN11*. The c.5C>T (p.Thr2Ile), NM_002834.5 mutation leads to a substitution of threonine (Thr) with isoleucine (Ile) at position 2. This amino acid change occurs in the N-terminal region of the PTPN11 protein. Similar to other pathogenic variants in *PTPN11*, this mutation is associated with RASopathies, potentially including Noonan syndrome or related disorders.

Sample 31 presented two distinct potentially pathogenic variants in different genes and a copy number variation. A heterozygous c.286A>G (p.Asn96Asp) variant was identified in *ABCA4* (NM_000350.3), a gene associated with Stargardt disease 1, a recessive macular dystrophy. Additionally, a heterozygous c.1226A>G (p.Asn409Ser) variant was found in *GBA1* (NM_001005741.3), as also observed in Sample 29 and associated with Gaucher disease type 1. Furthermore, SNP array analysis revealed a de novo duplication of approximately 15.7 Mb in the 2q31.1-q32.1 region. The clinical significance of this combination of findings, including the potential for compound heterozygosity in *ABCA4* (given the potentially pathogenic nature of the identified variant and the recessive inheritance of Stargardt disease), and the impact of the *GBA1* variant in conjunction with the CNV, requires further investigation and correlation with the proband’s phenotype and family history. The mother was found heterozygous for *ABCA4* and *GBA1* variants.

Sample 32 presented a pathogenic variant in *PAH*. The c.143T>C (p.Leu48Ser), NM_000277.3 mutation results in a substitution of leucine (Leu) with serine (Ser) at position 48 in the phenylalanine hydroxylase enzyme. Mutations in *PAH* are the cause of phenylketonuria (PKU), an autosomal recessive metabolic disorder. NGS analysis confirmed the heterozygous presence of this mutation in the proband, with the father also carrying the same heterozygous mutation.

Sample 33 presented a likely pathogenic variant in *PAH*. The c.688G>A (p.Val230Ile), NM_000277.1 mutation leads to a substitution of valine (Val) with isoleucine (Ile) at position 230 in the phenylalanine hydroxylase enzyme. Similar to other pathogenic variants in *PAH*, this mutation is associated with phenylketonuria (PKU). NGS analysis confirmed the heterozygous presence of this mutation in the proband, with the mother carrying the same heterozygous mutation.

Sample 34 presented a pathogenic variant in *PTPN11*. The c.1504C>T (p.Arg502Trp), NM_001330437.2 mutation results in a substitution of arginine (Arg) with tryptophan (Trp) at position 502. This amino acid change occurs within the phosphatase domain of the PTPN11 protein and is a known pathogenic variant associated with Noonan syndrome.

Sample 35 presented a pathogenic variant in *MYBPC3*. The c.927-9G>A, NM_000256.3 intronic variant affects a splice site in the *MYBPC3* gene, which encodes myosin-binding protein C, cardiac. This protein is a component of the cardiac muscle sarcomere. Splice site mutations in *MYBPC3* can lead to abnormal mRNA splicing, resulting in a non-functional or truncated protein. This type of mutation is a common cause of hypertrophic cardiomyopathy (HCM), an autosomal dominant disorder characterized by thickening of the heart muscle. NGS analysis confirmed the heterozygous presence of this mutation in the proband, with the mother also carrying the same heterozygous mutation.

Sample 36 presented a pathogenic variant in *GBA1*. The c.1448T>C (p.Leu483Pro), NM_001005741.3 mutation results in a substitution of leucine (Leu) with proline (Pro) at position 483 in the glucocerebrosidase enzyme. As mentioned previously for Sample 29, this enzyme is responsible for the breakdown of glucocerebroside, and mutations in GBA1 are a common cause of Gaucher disease type 1, a lysosomal storage disorder. NGS analysis confirmed the heterozygous presence of this mutation in the proband, and further analysis indicated that both parents were heterozygous for this mutation.

Chromosome 22 deletion was detected in Sample 6. The SNP array confirmed a deletion spanning the 22q11.2 region. The newborn was affected by DiGeorge syndrome, characterized by immunodeficiency, cardiac defects, palatal abnormalities, and hypoparathyroidism. In case 7, chromosome 4 deletion was identified, affecting the 4p16.3 region, associated with Wolf-Hirschhorn syndrome. SNP-array confirmed the deletion of 1.2Mb on chromosome 4 and only for this sample the placenta was available because of discrepancy between NIPS and SNP-array, the latter identified a duplication belonging to the placenta as described in [17]. Analysis of sample 10 revealed duplication of regions included in the short arm of chromosome 5 (5p), also known as “5p duplication syndrome”. This chromosomal abnormality is characterized by the duplication of a segment of the 5p arm, which often includes the distal 5p15 region. Analysis of sample 14 identified two distinct mutations in the *PAH* gene. The fetus inherited one mutation from each parent, resulting in a compound heterozygous state. The mutation, c.533A>G NM_000277.3, was inherited from the mother which causes the substitution of glutamic acid (Glu) with glycine (Gly) at position 178 in the enzyme. This change from a negatively charged amino acid to a neutral one can significantly impair the enzyme’s structure and function, potentially reducing its ability to metabolize phenylalanine. The second mutation, c.1208C>T NM_000277.3, was inherited from the father. This mutation causes the substitution of alanine (Ala) with valine (Val) at position 403. Although this involves a substitution between two nonpolar amino acids, even minor changes in the enzyme’s structure can affect its stability and function, contributing to a reduction in enzymatic activity necessary to prevent phenylalanine accumulation. In a compound heterozygous state, with different mutations on each allele of the *PAH* gene, the fetus is highly likely to develop PKU unless treated early in life. Analysis of samples 21 and 31 revealed, respectively, a deletion spanning approximately 21 Mb on the long arm of chromosome 13, and a duplication on the long arm of chromosome 2 spanning approximately 15.7 Mb.

### 3.3. Variant Overlap and Coverage Influence 

Table 2 and Table 3 present a comparison of SNP data between plasma samples, analyzed using a low-frequency sensitive variant caller, and the genetic data obtained from the amniotic fluid or born child, utilizing the GATK and ISAAC pipelines, respectively. The results showed in Table 2 and Table 3 report the percentages of common and unique variants identified in both the plasma samples and the child/fetus genetic profile. The proportion of common variants generally reflects the reliability of cfDNA analysis in detecting well-established genetic markers. In our analysis, the percentage of overlap for father-inherited variants and de novo mutations is 86% and 81% on average, respectively, when using GATK. Conversely, although ISAAC identifies a higher absolute number of variants, it shows a lower on average overlap, with 76% for father-inherited variants and 71% for de novo mutations. A filter for the coverage of non-overlapping variants, with a threshold set above 5 reads has been applied. Following this filtering, the percentage of overlapping sites increases overall for both variant callers up to 95%.

**Figure 3:**
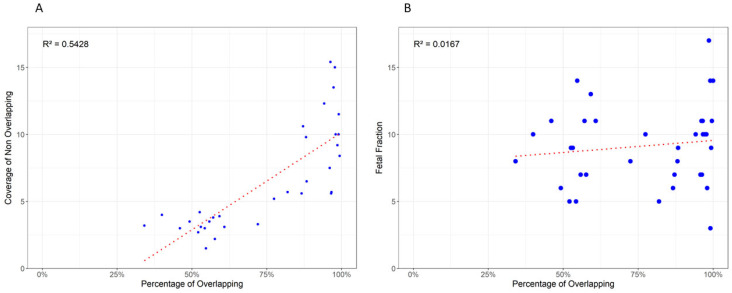
Panel (**A**) presents a dot plot depicting the relationship between the percentage of overlapping sites between cffDNA and the child’s genetic data and the coverage of non-overlapping sites. Panel (**B**) illustrates the relationship between the percentage of non-overlapping sites and the fetal fraction of the samples.

This coverage-dependent overlap between the two data sets is further illustrated in Figure 3, where the correlation coefficients (r^2^) for fetal fraction and coverage of non-overlapping variants are reported. The results indicate that the overlap is more strongly correlated with coverage (r^2^ = 0.54) than with fetal fraction (r^2^ = 0.0167).

## 4. Discussion

This study describes the development and preliminary validation of a 1069-gene panel aimed at the non-invasive detection of approximately 1000 monogenic disorders and copy number variations (CNVs). This work was conducted as a proof of principle, evaluating both the performance of the gene panel and a novel bioinformatics method specifically developed to detect microdeletions and microduplications smaller than one megabase. The genetic panel and the associated bioinformatics pipeline provide three key advantages: broad-spectrum analysis, advanced detection accuracy, and an optimized testing process.

### 4.1. Broad-Spectrum Analysis

The design of the panel allows for the simultaneous screening of a wide range of genetic conditions, including both common and rare disorders. These include Noonan spectrum disorders, skeletal disorders, craniosynostosis syndromes, Cornelia de Lange syndrome, Alagille syndrome, tuberous sclerosis syndrome, epileptic encephalopathy, SYNGAP1-related intellectual disability, CHARGE syndrome, Sotos syndrome, and Rett syndrome. These disorders are categorized into major clinical groups, such as Skeletal Dysplasias (e.g., analysis of genes like *ACAN*, *ACP5*, and *ACVR1*), Osteogenesis Imperfecta (*COL1A1*, *COL1A2*, *BMP1*), Metabolic Disorders (*ACE*, *AGT*, *BICC1*), and Cardiomyopathies, among others. One of the major advantages of this approach is its cost-effectiveness, which stems from its ability to analyze multiple categories of disorders simultaneously within a single, streamlined workflow, although an economic analysis is out of the scope of this work. The capacity to screen a broad spectrum of conditions in a high-throughput manner significantly optimizes resource utilization, making it particularly beneficial in cases where a detailed family history or prenatal imaging data is not yet available, such as in early pregnancies. The state-of-the-art research focused on the detection of the paternal inherited pathogenic variants both for recessive and dominant disorders and the analysis available to date is based on small custom genetic panels [18,19,20].

A notable innovation in this study is the bioinformatics pipeline specifically developed to complement the gene panel. This approach has the purpose to reach the detection also of CNVs, including microdeletions and microduplications, smaller than one megabase. This level of detail enables us not only to identify regions of duplication or deletion but also to detect partial aberrations where the entire region may not be affected.

These capabilities were validated through the identification of clinically relevant CNVs, such as those associated with DiGeorge syndrome and Wolf-Hirschhorn syndrome. The whole workflow has been validated through a comparison of the fetal data with the samples representing the fetus/child (AF, CVS, newborn). The gestational weeks, crucial for timing in prenatal diagnostics, were correlated with the detection efficiencies observed even if in the described study the detection accuracy was found strictly related to the coverage of the sample. The coverage is influenced by several aspects such as the biological sample quality, the library purity and so on [21], for this reason we introduced two filters, such as coverage > 5 reads and filtering of encode black list [13], in data analysis that ensure the filtering of artifacts.

Furthermore, deep sequencing ensures the reliable detection of low-frequency variants, even in challenging samples with low fetal fractions or high maternal DNA contamination. The combination of advanced genetics and computational tools underscores the feasibility, applicability and robustness of the proposed approach, even when implemented on a small cohort.

The targeted nature of the gene panel reduces the complexity of the test compared to whole-exome or whole-genome sequencing, enabling faster analysis and interpretation with a reduced turnaround time with possible positive implications in pregnancy management. This streamlined approach reduces costs while maintaining analytical depth, making comprehensive genetic screening more accessible also for low-risk population. The dual focus on detecting both single nucleotide variants and structural changes ensures that no clinically significant findings are overlooked.

### 4.2. Clinical Implications in Early Pregnancy

Additional advantage of this approach is its applicability in first-trimester pregnancies, often before any detailed prenatal imaging or clinical indications are available. In fact, for the majority of pregnancies, there are no clear clinical markers during the early weeks, which can delay the identification of potential genetic issues. In our cohort the majority of pregnancy had no indications to undergo a prenatal genetic screening. This test offers a valuable opportunity to derive actionable genetic information at a stage when interventions are still feasible. By highlighting genetic variants that could impact fetal development, the test can guide closer monitoring of specific parameters and provide a foundation for tailored pregnancy management. This early insight can influence decision-making, optimize care pathways, and potentially improve pregnancy outcomes by identifying cases that warrant additional surveillance or intervention.

This work serves as a proof of principle. Notably, some conditions—and therefore some of the genes included in our panel, such as *CFTR*, *GJB2*, and *HBB*—are relatively common, which increases the likelihood of detecting carriers. In many cases, identifying a simple carrier status permits further segregation studies in family members. Moreover, by including genes associated with high-impact conditions like cardiomyopathies, our approach also allows for the identification of affected parents through fetal testing. A key strength of our study is the implementation of a trio analysis, which enhances the interpretative power of our findings. Because to detect maternal variant is challenging, in our dataset the variant frequency suggests that the specific mutation could be carried by both the fetus and the mother. 

While the results demonstrate the functionality and potential of the genetic panel and bioinformatics method, the small sample size (36 families) limits the ability to derive robust generalizable statistics also because there are several variables that could influence the sample quality and of course the desired output. As mentioned before throughout the text the sample purity and integrity, the fetal fraction, the library quality and the sequencing coverage are all confounding factor that could potentially reduce the detection power of the workflow [22]. Metrics such as sensitivity, specificity, and predictive values cannot be reliably calculated at this stage. Larger cohort studies will be required to validate the approach further and establish reliable quantitative metrics for clinical use.

The ability to detect microdeletions and microduplications smaller than one megabase represents a significant technical advancement. Obviously this test has to be considered part of a more complex workflow to ensure that we would not miss any variation. Cases 21 and 31, seemed to be false negatives but in the light of the whole process we were able to identify the CNVs with different technique, this underlines the need to perform more tests on this side to refine the CNV identification algorithm and try to extend the upper detection limit.

In conclusion, this proof-of-principle study demonstrates that the test is feasible and robust in a screening setting. However, as a screening tool, it requires further refinement. Additional samples are needed to improve detection power and accuracy, as well as to enable a more comprehensive assessment of its performance statistics.

## Figures and Tables

**Table 1:** Details on the “VERA Revolution” results and confirmatory tests conducted on the analyzed families.

ID	Gesta-tional Week	FF	Indic-ation for NIPS	NIPS Results	Vera Revolution Panel Results	Confirmatory Test							Disease
						Biological Sample	NGS Results	Karyotype	Array Results	ACMG Classification	Maternal DNA	Paternal DNA	
1	10 + 4	11	MA	WT,XY	*GJB2* NM_004004.6: c.35del p.(Gly12ValfsTer2)	AF	*GJB2* NM_004004.6: c.35del p.(Gly12ValfsTer2), c.35del p.(Gly12ValfsTer2)	46,XY	ND	P (PVS1, PP5, PM2)	*GJB2* NM_004004.6: c.35del p.(Gly12ValfsTer2)	GJB2 NM_004004.6: c.35del p.(Gly12ValfsTer2)	Deafness, autosomal recessive 1A
2	11 + 5	7	MA	WT,XY	WT	AF	WT	46,XY	arr(X,Y)x1,(1-22)x2	NA	WT	WT	NA
3	15 + 2	11	AMA	dup(8)(p22q22.1), XY	WT	AF	WT	46, XY	arr(X,Y)x1,(1-22)x2, negative for UPD	NA	WT	WT	NA
4	14	10	MA	WT,XX	g.16(222,899_223,019dup);g.16(223,093_223, 625dup)	BS	WT	46,XX	arr[GRCh37] 16p13.3 (105,320-507,904)x3 *pat 410kb*; arr[GRCh37] 22q11.21-q11.22 (21,806,401-22,903,788)x1 *dn* 1.1 Mb	VUS/P	arr(X,1-22)x2	arr[GRCh37] 16p13.3 (105,320-507,904)x3	NA
5	13 + 6	9	MA	WT,XY	*CFTR* c.1521_1523del, p.Phe508del	AF	*CFTR* c.1521_1523del, p.Phe508del, c.1521_1523del, p.Phe508del	46,XY	ND	P (PS3, PM1, PM4, PP5)	c.1521_1523del, p.Phe508del	c.1521_1523del, p.Phe508del	Cystc fibrosis
6	12	8	AMA	WT, XY	g.22(18,222,123_18,222,243del);g.22(18,222,8 5_18,222,955del)	AF	NA	46,XY	arr[GRCh37] 22q11.21(18,877,787-21,462,353)x1 *dn*, 2.6 Mb	P	arr(X,1-22)x2	WT	DiGeorge syndrome
7	10 + 3	11	Previous pregnancy with IUGR	dup(4)(p16.3p12), XX	g.4(499476_499744del);g.4(500494_500614del)	AF	WT		arr[GRCh37] 4p16.3 (48,283- 1,243,573)x1 *dn*, 1.2Mb	P	arr(X,1-22)x2	arr(X,Y)x1,(1-22)x2	Wolf-Hirschhorn syndrome
8	12 + 6	14	MA	border trisomy 21, XY	WT	AF	NA	46,XX	arr(X,1-22)x2	NA	arr(X,1-22)x2	arr(X,Y)x1,(1-22)x2	
9	11 + 2	10	Congenital heart disease, suspicion of DiGeorge Syndrome	WT,XX	WT	AF	WT	46,XX	arr(1-22,X)x2, negative for UPD	NA	arr(X,1-22)x2	arr(X,Y)x1,(1-22)x2	NA
10	12 + 5	5	MA	dup(5)(p15.31p15.2), XX	g.5(6,602,533_6,602,653dup); g.5(6,604,260_6,60	AF	WT	46,XX,dup(5)(p15.32p15.1)	arr[GRCh37] 5p15.32 - p15.1(5.320.850-15.346.106)x3 *dn* 10Mb	P	arr(X,1-22)x2	arr(X,Y)x1,(1-22)x2	5p duplication syndrome
11	10 + 3	10	MA	WT,XY	*GJB2* NM_004004.6: c.35del p.(Gly12ValfsTer2)	AF	*GJB2* NM_004004.6: c.35del p.(Gly12ValfsTer2)	NA	NA	P (PVS1, PP5, PM2)	WT	*GJB2* NM_004004.6: c.35del p.(Gly12ValfsTer2)	Deafness, autosomal recessive 1A
12	13 + 5	9	cystic igroma	WT,XY	*RIT1* NM_006912.6: c.229G>T (p.Ala77Ser)	AF	*RIT1* NM_006912.6: c.229G>T (p.Ala77Ser)	46,XY	arr(X,Y)x1,(1-22)x2	P (PM5, PP5, PM1, PP3, PM2)	WT	WT	Noonan Symdrome
13	11 + 1	6	AMA	dup(22)(q11.21q12.1), XY	WT	AF	WT	46,XY	arr(X,Y)x1,(1-22)x2		arr(X,1-22)x2	arr(X,Y)x1,(1-22)x2	NA
14	12	6	MA	WT,XX	*PAH* NM_000277.3: c.533A>G (p.Glu178Gly), c.1208C>T (p.Ala403Val)	AF	*PAH* NM_000277.3: c.533A>G (p.Glu178Gly), c.1208C>T (p.Ala403Val)	46,XX	arr(X,1-22)x2	P (PP5, PM1, PM5, PP3, PM2)/P (PP5, PM1, PS3, BP4)	*PAH* NM_000277.1: c.533A>G (p.Glu178Gly)	*PAH* NM_000277.1: c.1208C>T (p.Ala403Val)	Phenylchetonuria
15	11	14	Increased NT, previous pregnancy with 15q11.2 deletion	WT,XX	WT	AF	WT	46,XX	arr(X,1-22)x2	NA	arr(X,1-22)x2	arr(X,Y)x1,(1-22)x2	NA
16	13 + 1	7	Increased NT	WT, XX	WT	AF	WT	46,XX	arr(X,1-22)x2	NA	arr(X,1-22)x2	arr(X,Y)x1,(1-22)x2	
17	12 + 5	17	Increased NT	WT,XX	*DHCR7* NM_001360.2 c.964-3C>G	CVS	*DHCR7* NM_001360.2 c.964-3C>G	46,XX	arr(X,1-22)x2	VUS (PP3, PM2)	WT	*DHCR7* NM_001360.2 c.964-3C>G	Smith-Lemli-Opitz Syndrome
18	12 + 2	14	Increased NT	WT,XX	WT	AF	NA	46,XX	arr(X,1-22)x2	NA	arr(X,1-22)x2	arr(X,Y)x1,(1-22)x2	NA
19	11	11	MA	Border trisomy 18, XX	WT	AF	NA	46,XX	arr(X,1-22)x2	NA	arr(X,1-22)x2	arr(X,Y)x1,(1-22)x2	NA
20	12	5	Increased NT	WT	WT	AF	NA			NA			NA
21	12 + 3	7	MA	del(13)(q21.2q31.2), XX	WT	AF	NA	46,XX,del(13)(q21.2q31.2)	arr[GRCh37] 13q21.2 - q31.1(60.932.642-81.657.010)x1 dn	NA	arr(X,1-22)x2	arr(X,Y)x1,(1-22)x2	NA
22	12 + 4	7	MA	WT,XY	*GJB2* NM_004004.6: c.109G>A p.(Val37Ile); c.583A>G, p.(Met195Val)	AF	NA	46,XY	*GJB2* NM_004004.6: c.109G>A p.(Val37Ile); c.583A>G, p.(Met195Val)	P (PM1, PM5, PP3, PP5, PM2)/P (PM5, PM1, PP3, PM2, PP5)	*GJB2 NM_004004.6:* c.109G>A p.(Val37Ile)	*GJB2 NM_004004.6*: c.583A>G, p.(Met195Val)	Deafness, autosomal recessive 1A
23	12	5	PMA	trisomy 3, XX	WT	AF	NA	46,XX	arr(X,1-22)x2	NA	arr(X,1-22)x2	arr(X,Y)x1,(1-22)x2	NA
24	9	10	WT	WT	WT	BS	NA	46,XY	arr(X,Y)x1,(1-22)x2	NA	arr(X,1-22)x2	arr(X,Y)x1,(1-22)x2	NA
25	14 + 5	10	NA	WT	*TCOF1* NM_001371623.1:c.1999dup, (p.Arg667fs)	AF	*TCOF1* NM_001371623.1: c.1999dup, (p.Arg667fs)	46,XY	arr(X,Y)x1,(1-22)x2	P (PVS1, PM2, PP5)	arr(X,1-22)x2	arr(X,Y)x1,(1-22)x2	Treacher Collins
26	10 + 3	8	AMA	WT,XY	*PTPN11* NM_002834.5 c.1471C>T p.(Pro491Ser)	AF	*PTPN11* NM_001330437.2 c.1483C>T p.(Pro495Ser)	46,XY	arr(X,Y)x1,(1-22)x2	P (PP5, PM5, PM1, PP3, PM2)	WT	WT	RASopathy
27	12 + 3	5	MA	WT,XY	*MCCC2* NM_022132.5 c.1015G>A, p.(Val339Met)	AF	*MCCC2* NM_022132.5 c.1015G>A, p.(Val339Met)	46,XY	arr(X,Y)x1,(1-22)x2	P (PP5, PM1)	MCCC2 NM_022132.5 c.1015G>A, p.(Val339Met)	MCCC2 NM_022132.5 c.1015G>A, p.(Val339Met)	3-Methylcrotonyl-CoA carboxylase 2 deficiency
28	11 + 1	12	MA	WT,XY	*GJB2* NM_004004.6: c.35del p.(Gly12ValfsTer2)	BS	*GJB2* NM_004004.6: c.35del p.(Gly12ValfsTer2)	46,XY	arr(X,Y)x1,(1-22)x2	p (PVS1, PP5, PM2)	WT	*GJB2* NM_004004.6: c.35del p.(Gly12ValfsTer2)	Deafness, autosomal recessive 1A
29	13 + 3	20	MA	WT,XY	*GBA1* NM_001005741.3: c.1226A>G (p.Asn409Ser)	BS	*GBA1* NM_001005741.3: c.1226A>G (p.Asn409Ser)	46,XY	arr(X,Y)x1,(1-22)x2	P (PS3, PM1, BP4)	*GBA1* NM_001005741.3: c.1226A>G (p.Asn409Ser)	WT	Gaucher disease type I (GD1)
30	12 + 2	6		WT,XY	*PTPN11* NM_002834.5 c.5C>T, p.(Thr2Ile)	AF	PTPN11 NM_002834.5 c.5C>T, p.(Thr2Ile)	46,XY	arr(X,Y)x1,(1-22)x2	P (PP5, PM2, PP2, BP3)	WT	WT	RASopathy
31	12 + 6	11	AMA	dup(2)(q31.1q32.1), XX	*ABCA4* NM_000350.3 c.286A>G, p.(Asn96Asp), *GBA1* NM_001005741.3 c.1226A>G, p.(Asn409Ser)	AF	ABCA4 NM_000350.3 c.286A>G, p.(Asn96Asp), *GBA1* NM_001005741.3 c.1226A>G, p.(Asn409Ser)	46,XX	arr[GRCh37] 2q31.1 - q32.1 (173.278.777-188.970.013)x3 dn, 15.7 Mb	P/PL (PM5, PP5, PM1, PP3, PM2), P/PL (PS3, PM1, BP4)	WT	ABCA4 NM_000350.3 c.286A>G, p.(Asn96Asp), *GBA1* NM_001005741.3 c.1226A>G, p.(Asn409Ser)	Stargardt disease 1, Gaucher disease type I (GD1)
32	11 + 3	12	AMA	WT,XY	*PAH* NM_000277.3, c.143T>C p.(Leu48Ser)	BS	*PAH* NM_000277.3, c.143T>C p.(Leu48Ser)	46,XY	arr(X,Y)x1,(1-22)x2	P (PP5, PP3, PM1, PS3, PM2)	WT	*PAH* NM_000277.1, c.143T>C p.(Leu48Ser)	Phenylchetonuria
33	10 + 4	9	AMA	WT,XY	*PAH* NM_000277.1, c.688G>A p.(Val230Ile)	BS	*PAH* NM_000277.1, c.688G>A p.(Val230Ile)	46,XY	arr(X,Y)x1,(1-22)x2	LP (PP5, PM1, PM5, PS3, PM2, BP4	*PAH* NM_000277.1, c.688G>A p.(Val230Ile)	WT	Phenylchetonuria
34	11 + 4	15	AMA	WT,XY	*PTPN11* NM_001330437.2:c.1504C>T, p.(Arg502Trp)	AF	*PTPN11* NM_001330437.2:c.1504C>T, p.Arg502Trp	46,XY	arr(X,Y)x1,(1-22)x2	P (PM1, PM5, PP3, PM2)	WT	WT	RASopathy
35	12 + 0	11	MA	WT,XX	*MYBPC3* NM_000256.3 c.927-9G>A	AF	*MYBPC3* NM_000256.3 c.927-9G>A	46,XX	arr(X,1-22)x2	P (PP5, PP3, PM2)	WT	MYBPC3 NM_000256.3 c.927-9G>A	Hypertrophic cardiomyopathy
36	12 + 0	18	MA	WT,XX	*GBA1* NM_001005741.3 c.1448T>C, p.(Leu483Pro)	AF	*GBA1* NM_001005741.3 c.1448T>C, p.(Leu483Pro)	46,XX	arr(X,1-22)x2	P (PP3. PP5, PM1, PM5, PM2)	*GBA1* NM_001005741.3 c.1448T>C, p.(Leu483Pro)	*GBA1* NM_001005741.3 c.1448T>C, p.(Leu483Pro)	Gaucher disease type I (GD1)

AF: amniotic fluid; BS: buccal swab; FF: Fetal fraction; NA: Not Applicable; ND: Not Done; WT: Wild Type.

**Table 2:** The table shows the results of the comparison between variant data from plasma, identified using a low-frequency sensitive variant caller, and data from the born child, identified using GATK.

		Father-Inherited					De Novo			
ID	Total Variants	Common Variants	Uncommon Variants	% of Common	Coverage of Uncommon	Total Variants	Common Variants	Uncommon Variants	% of Common	Coverage of Uncommon
1	3448	1633	1815	47%	3.8	6754	2446	4308	36%	2.2
2	559	487	72	87%	10.6	309	309	0	100%	19.3
3	460	444	16	96%	5.6	272	272	0	100%	15.8
4	2682	1583	1099	59%	4.0	4038	1613	2425	40%	1.6
5	3652	1920	1732	52%	4.2	6139	2018	4121	33%	1.6
6	2700	1256	1444	46%	3.2	4656	1587	3069	34%	1.7
7	1921	1379	542	72%	3.1	1838	1118	720	61%	1.4
8	530	525	5	99%	11.5	343	341	2	99%	7.0
9	448	438	10	98%	15.0	320	320	0	100%	12.8
10	164	89	75	54%	3.0	680	476	204	70%	3.3
11	500	483	17	96%	5.7	204	204	0	100%	8.8
12	468	465	3	99%	8.4	301	258	43	86%	6.9
13	464	402	62	87%	5.6	288	288	0	100%	4.5
14	2720	1605	1115	59%	3.5	4383	1535	2848	35%	1.4
15	426	233	193	55%	1.5	1832	231	1601	13%	0.9
16	458	441	17	96%	15.4	405	287	118	71%	3.3
17	498	491	7	99%	9.2	290	290	0	100%	7.2
18	478	478	0	100%	21.0	304	304	0	100%	3.7
19	490	488	2	99%	18.4	258	258	0	100%	24.4
20	410	336	74	82%	5.7	3222	262	2960	8%	0.8
21	470	271	199	56%	2.2	221	147	74	67%	2.5
22	6586	1958	4628	30%	3.5	3214	2112	1102	66%	1.5
23	478	249	229	52%	2.7	315	240	75	76%	3.7
24	1952	1511	441	77%	5.2	1952	1511	441	77%	1.9
25	481	453	28	94%	12.3	318	318	0	100%	9.9
26	610	535	75	88%	9.8	355	350	5	99%	17.1
27	2900	1700	1200	59%	3.9	5000	1800	3200	36%	2.0
28	430	380	50	88%	6.5	260	255	5	98%	5.2
29	580	565	15	97%	13.5	330	330	0	100%	11.0
30	2500	1150	1350	46%	3.0	4500	1500	3000	33%	1.8
31	1800	1300	500	72%	3.3	1700	1050	650	62%	1.3
32	495	490	5	99%	10.0	310	308	2	99%	6.8
33	450	440	10	98%	16.0	300	300	0	100%	13.0
34	150	80	70	53%	3.1	700	490	210	70%	3.5
35	510	490	20	96%	7.5	210	210	0	100%	9.0
36	470	460	10	98%	10.0	295	250	45	85%	7.1

**Table 3:** Results of the comparison between variant data from plasma, identified using a low-frequency sensitive variant caller, and data from the born child called with ISAAC.

		Father-Inherited					De Novo			
ID	Total Variants	Common Variants	Uncommon Variants	% of Common	Coverage of Uncommon	Total Variants	Common Variants	Uncommon Variants	% of Common	Coverage of Uncommon
1	3448	1633	1815	47%	3.8	6754	2446	4308	36%	2.2
2	559	487	72	87%	10.6	309	309	0	100%	19.3
3	460	444	16	96%	5.6	272	272	0	100%	15.8
4	2682	1583	1099	59%	4.0	4038	1613	2425	40%	1.6
5	3652	1920	1732	52%	4.2	6139	2018	4121	33%	1.6
6	2700	1256	1444	46%	3.2	4656	1587	3069	34%	1.7
7	1921	1379	542	72%	3.1	1838	1118	720	61%	1.4
8	530	525	5	99%	11.5	343	341	2	99%	7.0
9	448	438	10	98%	15.0	320	320	0	100%	12.8
10	164	89	75	54%	3.0	680	476	204	70%	3.3
11	500	483	17	96%	5.7	204	204	0	100%	8.8
12	468	465	3	99%	8.4	301	258	43	86%	6.9
13	464	402	62	87%	5.6	288	288	0	100%	4.5
14	2720	1605	1115	59%	3.5	4383	1535	2848	35%	1.4
15	426	233	193	55%	1.5	1832	231	1601	13%	0.9
16	458	441	17	96%	15.4	405	287	118	71%	3.3
17	498	491	7	99%	9.2	290	290	0	100%	7.2
18	478	478	0	100%	21.0	304	304	0	100%	3.7
19	490	488	2	99%	18.4	258	258	0	100%	24.4
20	410	336	74	82%	5.7	3222	262	2960	8%	0.8
21	470	271	199	56%	2.2	221	147	74	67%	2.5
22	6586	1958	4628	30%	3.5	3214	2112	1102	66%	1.5
23	478	249	229	52%	2.7	315	240	75	76%	3.7
24	1952	1511	441	77%	5.2	1952	1511	441	77%	1.9
25	481	453	28	94%	12.3	318	318	0	100%	9.9
26	610	535	75	88%	9.8	355	350	5	99%	17.1
27	2900	1700	1200	59%	3.9	5000	1800	3200	36%	2.0
28	430	380	50	88%	6.5	260	255	5	98%	5.2
29	580	565	15	97%	13.5	330	330	0	100%	11.0
30	2500	1150	1350	46%	3.0	4500	1500	3000	33%	1.8
31	1800	1300	500	72%	3.3	1700	1050	650	62%	1.3
32	495	490	5	99%	10.0	310	308	2	99%	6.8
33	450	440	10	98%	16.0	300	300	0	100%	13.0
34	150	80	70	53%	3.1	700	490	210	70%	3.5
35	510	490	20	96%	7.5	210	210	0	100%	9.0
36	470	460	10	98%	10.0	295	250	45	85%	7.1

## Data Availability

Protocols and de-identified, aggregated data that underlie the results reported in this article are available for non-commercial scientific purposes upon reasonable request from the corresponding author.

## Supplementary Materials

The following supporting information can be downloaded at https://www.mdpi.com/article/10.3390/genes16040427/s1, Table S1: Complete list of gene panel; Table S2: Coordinates of the regions covered by gene panel for CNV detection.
